# Cerebral Amyloid Angiopathy-Related Inflammation Mimicking Posterior Reversible Encephalopathy Syndrome: A Case Report of Subacute Gerstmann Syndrome in an Elderly Patient

**DOI:** 10.7759/cureus.107318

**Published:** 2026-04-18

**Authors:** Nino Abesadze, Jana Pavlickova

**Affiliations:** 1 Medicine, First Faculty of Medicine, Charles University, Prague, CZE; 2 Neurology, First Faculty of Medicine, Charles University and General Hospital, Prague, CZE

**Keywords:** antinuclear antibodies (ana), autoinflammation, borreliosis, caa-related inflammation (caa-ri), cerebral amyloid angiopathy, gerstmann syndrome, posterior reversible encephalopathy syndrome (pres), vasogenic brain edema

## Abstract

Cerebral amyloid angiopathy-related inflammation (CAA-RI) is a rare immune-mediated complication of cerebral amyloid angiopathy in elderly patients and may closely mimic posterior reversible encephalopathy syndrome (PRES) both clinically and radiologically, making diagnosis challenging. Even though there is an apparently favorable initial outcome after steroid or immunosuppressive treatment, the CAA-RI course is unpredictable and may be associated with relapse or unfavorable neurological outcomes. We report a 76-year-old woman who presented with alexia, acalculia, mild right hemiparesis, and diplopia, consistent with a partially expressed Gerstmann syndrome. Cerebrospinal fluid analysis showed elevated protein without pleocytosis, while the electroencephalography (EEG) demonstrated focal slowing. Serological testing revealed antinuclear antibody (ANA) positivity and Borrelia burgdorferi IgG seropositivity in serum without evidence of intrathecal antibody synthesis. After exclusion of infectious, ischemic, and vasculitic etiologies, the diagnosis of probable CAA-RI was established according to the Boston criteria version 2.0, based on the clinical presentation and characteristic MRI findings, including white matter hyperintensities in a multispot pattern. High-dose intravenous corticosteroid therapy was initiated, resulting in marked clinical improvement. The diagnosis of CAA-RI was supported by the overall patient presentation, characteristic imaging findings, exclusion of alternative etiologies, and response to immunosuppressive treatment. This case highlights the diagnostic challenge of distinguishing CAA-RI from PRES and emphasizes the importance of early recognition to enable timely treatment.

## Introduction

Cerebral amyloid angiopathy (CAA) is a small vessel disease characterized by amyloid-β deposition in cortical and leptomeningeal vessels, predisposing patients to lobar hemorrhage, cerebral microbleeds, and cognitive impairment [1]. It is commonly associated with aging and is frequently observed in patients with Alzheimer’s disease [2].

A rare inflammatory variant, CAA-related inflammation (CAA-RI), represents an immune-mediated response to vascular amyloid deposition and is associated with potentially reversible neurological deficits [3]. Clinically, CAA-RI presents with subacute cognitive decline, seizures, behavioral changes, and focal deficits. Neuroimaging typically demonstrates asymmetric cortico-subcortical white matter hyperintensities with vasogenic edema and multiple microbleeds [4]. 

Posterior reversible encephalopathy syndrome (PRES) may closely mimic CAA-RI, presenting with headache, seizures, visual disturbances, and altered mental status. Imaging typically shows posterior-predominant vasogenic edema, often symmetric and without microbleeds. PRES usually arises from endothelial injury due to hypertension, toxins, or cytokines and is often reversible with treatment, whereas CAA-RI requires immunosuppression [5]. CSF is generally nonspecific, and advanced imaging may help distinguish PRES from inflammatory or vasculopathic mimics. This distinction is important because treatment of PRES is typically supportive and directed at the underlying cause, whereas CAA-RI requires immunosuppressive therapy [6].

This case highlights CAA-RI presenting as a subacute Gerstmann syndrome with imaging features mimicking PRES, emphasizing the diagnostic challenge of differentiating CAA-RI from PRES in the setting of overlapping clinical and radiological findings. It further demonstrates how such overlap may delay diagnosis and illustrates the importance of considering CAA-RI in elderly patients presenting with focal cognitive syndromes and PRES-like imaging. This report aims to highlight CAA-RI as a PRES mimic and to emphasize the importance of applying structured diagnostic criteria to distinguish these entities and guide appropriate immunosuppressive treatment.

## Case presentation

A 76-year-old woman presented to the neurology department with features consistent with Gerstmann syndrome, including finger agnosia, agraphia, acalculia, and right-left disorientation. She also exhibited mild right-sided hemiparesis and reported horizontal diplopia without objective ocular motility impairment. The patient described a sudden onset of reading difficulty and impaired calculation abilities, followed over several days by progressive confusion and cognitive decline. As a result, she was admitted on Day 1 for further evaluation. She reported episodes of disorientation and difficulty recalling the reason for her hospitalization, but remained aware of her deficits and expressed distress regarding her memory lapses. 

Her medical history was unremarkable, with no known autoimmune disease, malignancy, cerebrovascular events, or recent infection. Routine blood tests demonstrated stable electrolytes, preserved renal function, and consistently low inflammatory markers. Liver enzyme levels were within the reference range, and thyroid function and HIV serology were normal.

Cerebrospinal fluid (CSF) (Table 1) analysis demonstrated elevated total protein (1.03 g/L) with normal cell counts, and the albumin quotient (13.13) indicated dysfunction of the blood-CSF barrier. Reiber diagram analysis suggested increased IgG and IgM indices with borderline elevation of IgA. However, oligoclonal band testing demonstrated identical bands in serum and CSF without CSF-restricted bands, arguing against typical multiple sclerosis-type intrathecal synthesis. Cytology was unremarkable, and microbiological cultures were sterile. Given the positive Borrelia serology in serum (elevated IgG and positive Western blot IgG), neuroborreliosis was carefully considered. However, the absence of intrathecal Borrelia antibody synthesis (antibody index <1.5 for both IgM and IgG) effectively excluded active central nervous system (CNS) Lyme disease. CSF PCR testing for Epstein-Barr virus (EBV), cytomegalovirus (CMV), herpes simplex virus (HSV)-1/2, varicella-zoster virus (VZV), and enteroviruses was negative. Parasitological testing following recent travel was unremarkable.

**Table 1:** CSF values and autoimmune panel ANA: antinuclear antibody, RF: rheumatoid factor, ENA: extractable nuclear antigen, ANCA: antineutrophil cytoplasmic antibodies, MPO: myeloperoxidase, PR3: proteinase 3

Test	Patient value	Normal range	Interpretation
CSF Leukocytes (cells/µL)	2	0-5	Normal
CSF Erythrocytes (cell/µL)	1	0	Traumatic/insignificant
Total CSF Protein (g/L)	1.03	0.15-0.45	Elevated
CSF Glucose (mmol/L)	3.26	2.2-4.4	Normal
Glucose Quotient	0.6	≥0.6	Normal
CSF Lactate (mmol/L)	1.63	1.2-2.1	Normal
CSF Albumin (mg/L)	516	0-350	Elevated
Albumin Quotient	13.13	<8 (age adjusted)	Blood-CSF barrier dysfunction
IgG Index	0.86	<0.7	Mild elevation
IgA Index	0.70	<0.6-0.7	Borderline elevation
IgM Index	0.59	<0.15	Elevated
Oligoclonal Bands	Identical in serum and CSF	No CSF-restricted bands	No intrathecal synthesis
ANA	1:320	<1:100	Positive
ANA Pattern	Granular	-	Nonspecific
RF (IU/mL)	<8.56	<14	Negative
Anti-dsDNA (IU/mL)	<9.8	<25	Negative
Anti-ENA panel	9.1	0-20	Negative
ANCA	Negative	Negative	Negative
Anti-MPO	<3.2	<5	Negative
Anti-PR3	<2.3	<5	Negative

Autoimmune screening revealed a positive antinuclear antibody (ANA) titer of 1:320 with a granular pattern (Table 1). The isolated ANA positivity, in the absence of systemic features or disease-specific autoantibodies, was considered nonspecific.

**Figure 1:**
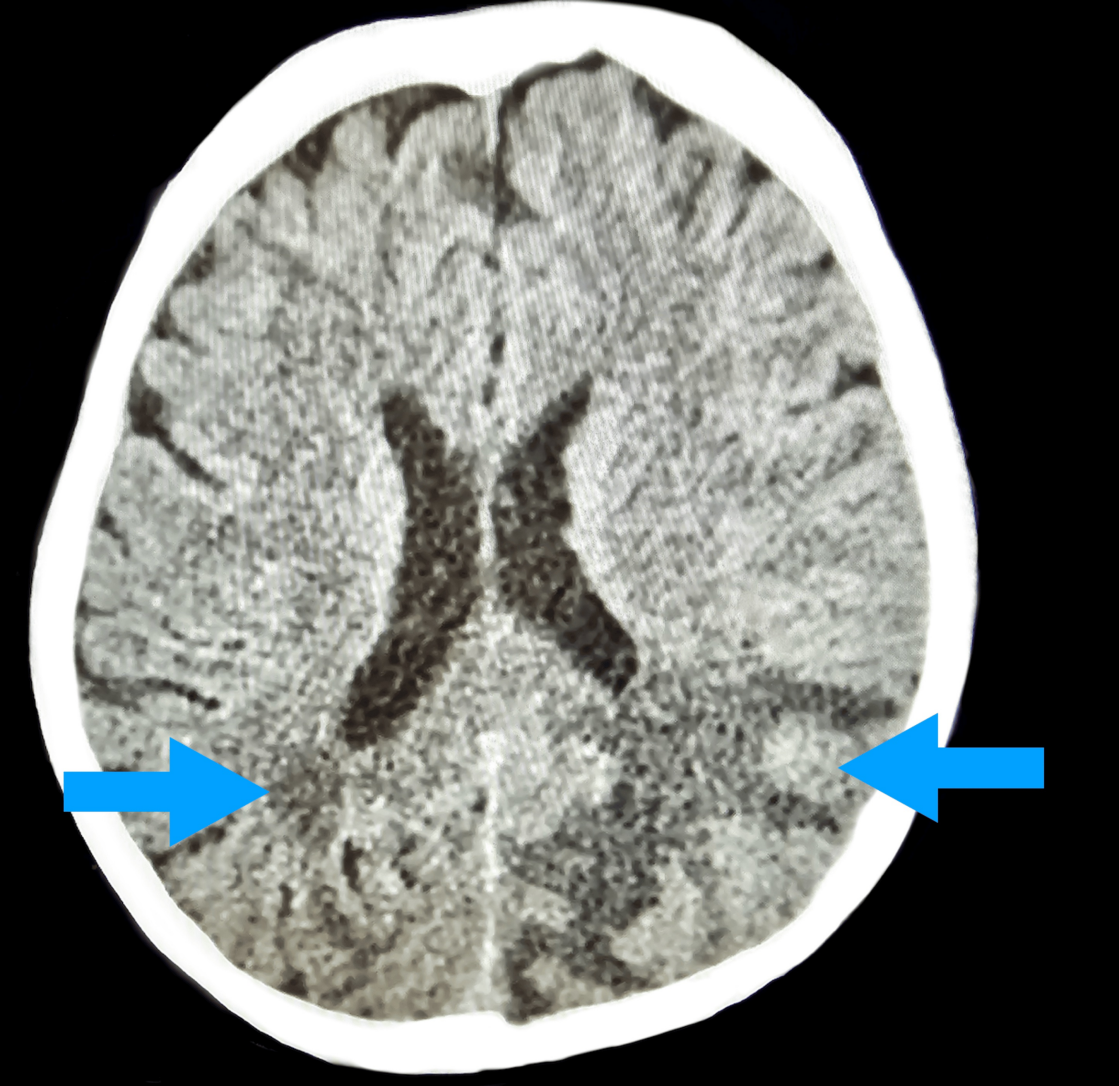
Axial brain CT demonstrating bilateral occipital hypodensities in the posterior white matter (blue arrows), consistent with vasogenic edema.

The clinical course followed a subacute progression, with admission on Day 1, initial CT imaging on Day 2, MRI on Day 18, and subsequent initiation of corticosteroid therapy after multidisciplinary evaluation. A brain CT performed on Day 2 (Figure 1) served as the initial imaging modality to exclude acute hemorrhage and large territorial ischemia. 

**Figure 2:**
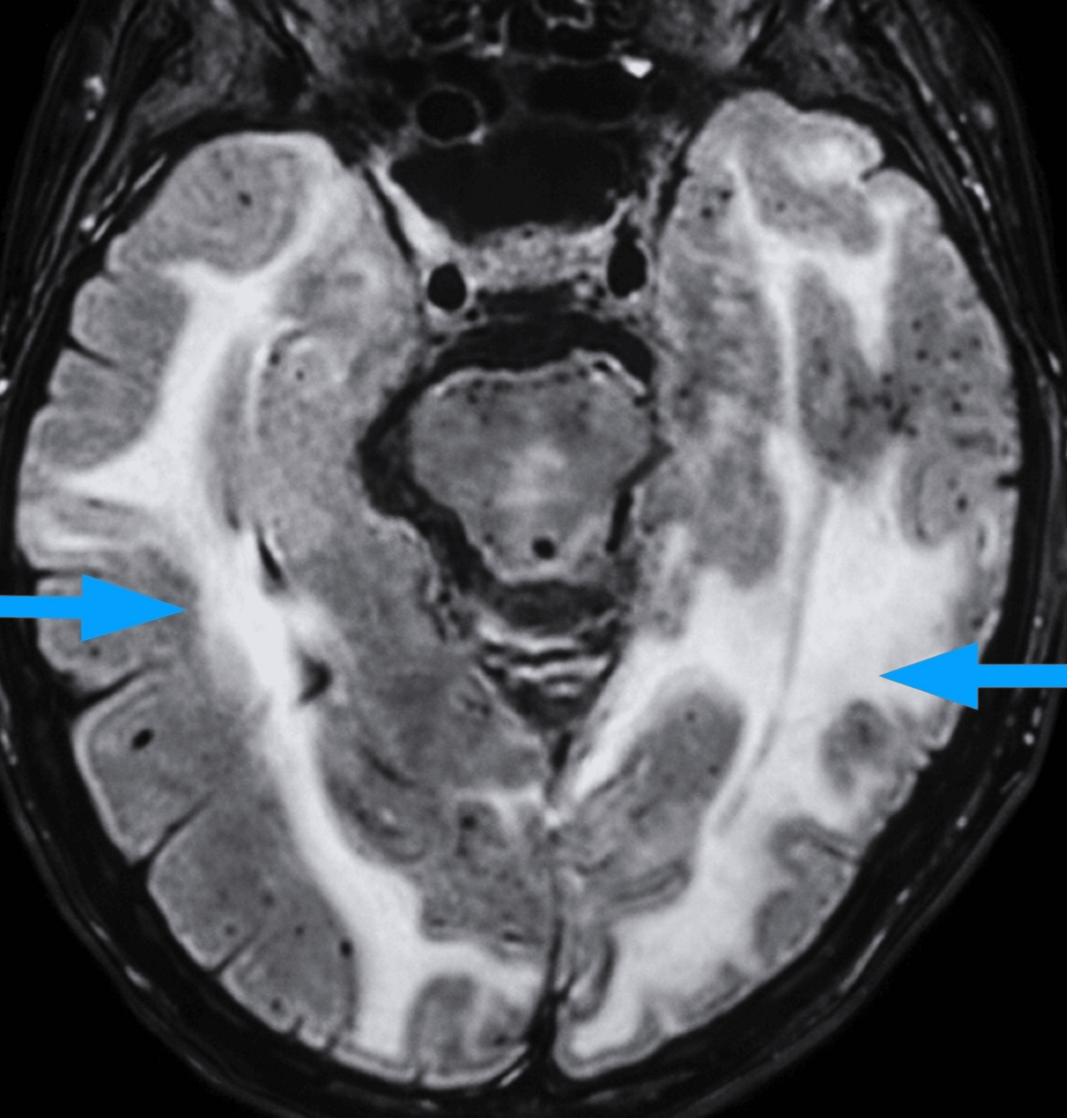
Axial fluid-attenuated inversion recovery (FLAIR) MRI showing asymmetric bilateral cortico-subcortical hyperintensities with posterior predominance, consistent with vasogenic edema (blue arrows).

**Figure 3:**
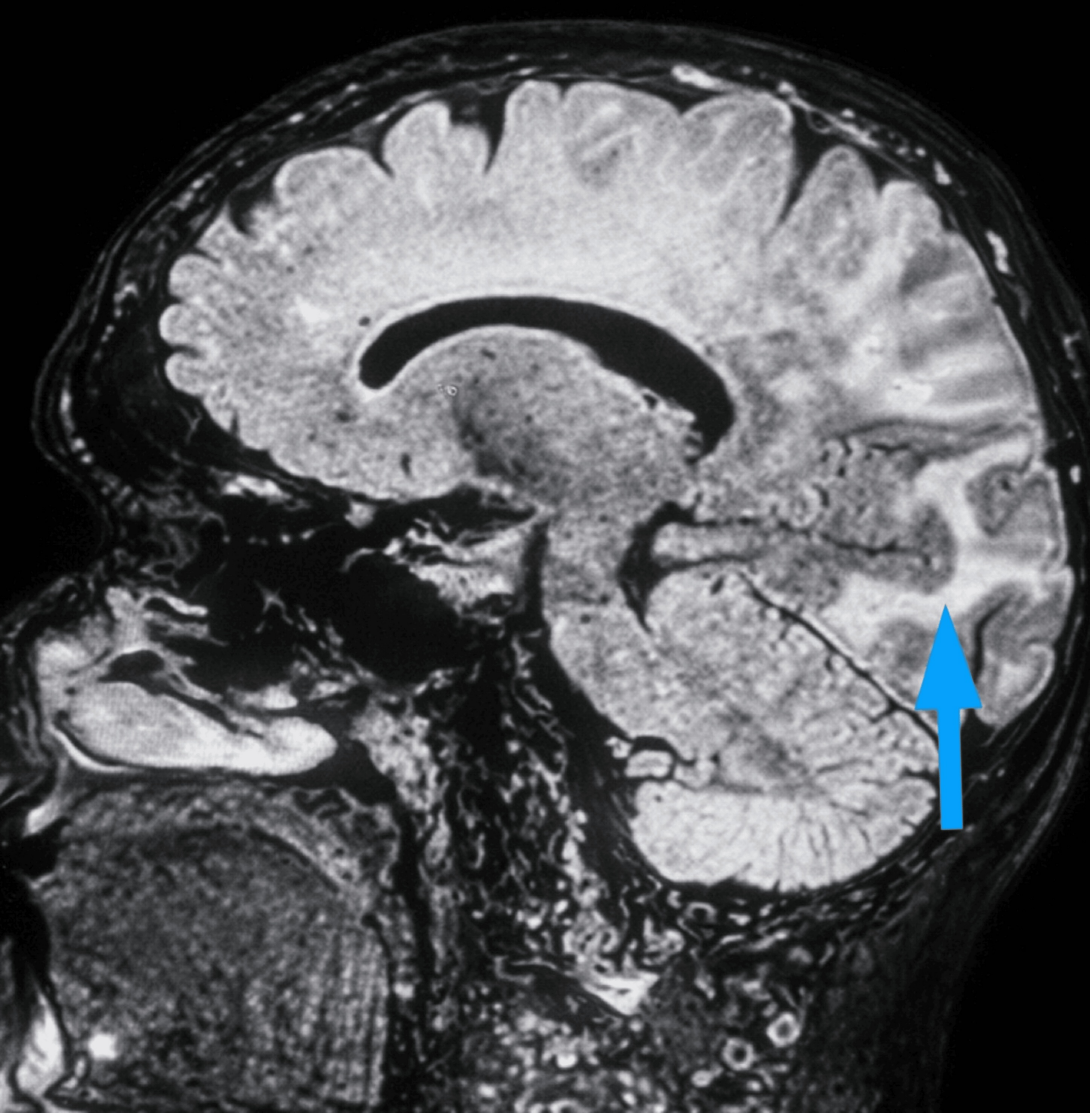
Sagittal fluid-attenuated inversion recovery (FLAIR) sequence demonstrating diffuse supratentorial white matter hyperintensities, most prominent in the parieto-occipital regions (blue arrow).

Given the subacute presentation, clinical stability, and absence of focal deterioration, advanced MRI was subsequently scheduled to further characterize the evolving white matter lesions and assess for inflammatory or vasculopathic etiologies. During this interval, the patient was managed conservatively without immunosuppressive therapy. A neurology consultation was obtained early in hospitalization, and serial neurological examinations were performed while awaiting MRI. Brain MRI on Day 18 (Figures 2, 3) demonstrated extensive confluent white matter lesions in all lobes, with a maximum in the bilateral parieto-occipital regions (more pronounced on the left), without diffusion restriction or contrast enhancement, consistent with vasogenic edema. Susceptibility-sensitive sequences revealed multiple lobar microbleeds, including periventricular involvement. The findings were considered consistent with CAA-RI, with PRES remaining in the differential diagnosis, and showed mild progression compared with imaging from Day 2.

**Figure 4:**
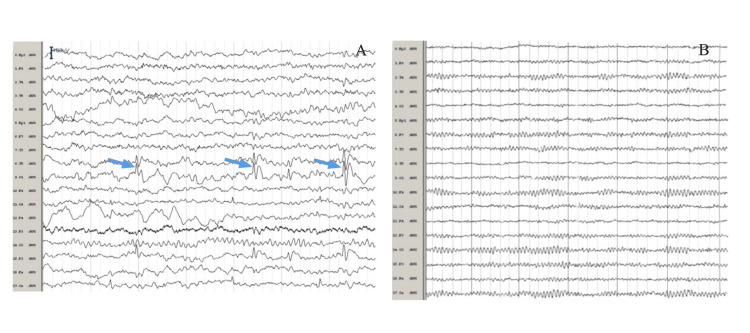
(A) Initial EEG demonstrating periodic discharges (blue arrows), consistent with focal cortical dysfunction. (B) Follow-up EEG after benzodiazepine challenge and initiation of lamotrigine, showing resolution of periodic discharges and improved background symmetry.

Electroencephalography (Figure 4) demonstrated focal slowing over the left hemisphere with periodic discharges; however, diagnostic criteria for non-convulsive status epilepticus were not fulfilled. A benzodiazepine challenge suppressed the pathological activity, after which antiepileptic therapy with lamotrigine was initiated, resulting in subsequent EEG improvement.

Following the initial assessment by the primary medical team, the patient underwent early evaluation by a neurology consultant during hospitalization. A differential diagnosis, including PRES, primary CNS vasculitis, and CAA-RI, was established. The decision to proceed with further MRI evaluation and to initiate high-dose corticosteroid therapy was reached after a multidisciplinary discussion led by the neurology service. The patient received intravenous methylprednisolone 500 mg/day for 10 days (cumulative dose 5 g), followed by a structured oral prednisone taper.

An MRI performed three months after hospitalization showed no abnormalities.

## Discussion

CAA-RI represents an inflammatory response to vascular amyloid deposition and is increasingly recognized as a reversible cause of subacute encephalopathy in elderly patients. The pathophysiology is thought to involve immune-mediated perivascular inflammation directed against amyloid-β [7].

There have been many ideas about why the autoimmune mechanism is activated in cases of severe CAA. Reported cases of CAA-RI have been described in association with diverse immune-mediated and inflammatory triggers, including systemic autoimmune diseases, infections, and vaccination. A summary of previously reported cases highlighting patient characteristics, potential risk factors, timing of presentation, treatment, and comorbid conditions is provided in Table 2.

**Table 2:** Published cases of cerebral amyloid angiopathy-related inflammation related to different risk factors. ANA: antinuclear antibody

Study	Patient (Age/Sex)	Risk Factor	Presentation	Comorbid Conditions	MRI Findings	Treatment and Response
Tang et al., 2024 [8]	67 / Male	SARS-CoV-2 vaccination	48 h	Chronic left hip osteomyelitis	Bilateral cortical and subcortical hyperintensities with progressive occipital microbleeds	Intravenous immunoglobulin (IVIG) for 5 days and intravenous methylprednisolone (1000 mg) for 3 days, followed by additional immunosuppressive therapy (cyclophosphamide). Delayed treatment response resulted in significant brain atrophy.
Seifert et al., 2023 [9]	61 / Female	Systemic autoimmune disease (granulomatosis with polyangiitis)	Subacute	Granulomatosis with polyangiitis; osteoporosis; depressive disorder; prior malnutrition	Pronounced frontotemporal vasogenic edema	Intravenous methylprednisolone 500 mg daily for 5 days (total 2,500 mg), followed by oral prednisolone tapered from 80 mg to 60 mg, 40 mg, and 20 mg at two-week intervals. Gradual clinical improvement observed.
Bozovic et al., 2023 [10]	65 / Male	High-titer ANA positivity	Subacute	Essential hypertension; prior myocardial infarction; calcified frontal meningioma	Hypointense lesions predominantly in the right frontotemporal and occipital regions	Intravenous methylprednisolone (1 g/day) for 5 days followed by oral prednisone (1 mg/kg) tapered over 3 months. Clinical improvement within 3 weeks.
Nelson et al., 2019 [11]	57 / Male	Disseminated mycobacterial infection during immunosuppression	Subacute	Cardiopulmonary sarcoidosis; dilated cardiomyopathy; orthotopic heart transplantation; chronic immunosuppression	Hyperintense lesions in the right temporal and parietal lobes	Intravenous methylprednisolone (1 g/day) for 5 days followed by oral prednisone 60 mg daily with gradual taper over 3 months. Radiological and clinical improvement within 1 month.

There is speculation that neuroinflammation in Lyme neuroborreliosis alters amyloid metabolism, with decreased CSF levels of α-sAPP, β-sAPP, and phosphorylated tau during acute infection that normalize after treatment [12]. These findings suggest a possible interaction between infection-related neuroinflammation and amyloid metabolism. However, the relationship between Borrelia infection and CAA-RI remains speculative, and no causal link has been established.

A prior study demonstrated the presence of Borrelia burgdorferi antigen and DNA in brain tissue from patients with Alzheimer-type pathology, including one individual with a history of Lyme disease. Borrelia-positive aggregates were shown to co-localize with amyloid-β and phospho-tau, and experimental infection models demonstrated increased expression of amyloid-β and phospho-tau, suggesting a potential mechanistic link between Borrelia infection and cerebral amyloidosis [13].

Previous reports have described ANA positivity in chronic-stage CAA-RI; the present case demonstrates a subacute presentation, suggesting that immune serological abnormalities may be observed across different disease stages [14].

While these associations have been described as influencing the autoimmune etiology of the disease, the bacterial and immune components have not yet been linked to causing the disease.

The present case highlights several diagnostic challenges due to the substantial clinical and radiological overlap between CAA-RI and PRES. Initial neuroimaging demonstrated vasogenic edema without diffusion restriction, closely resembling PRES; however, the presence of numerous cerebral microbleeds and the asymmetric distribution of lesions pointed toward an underlying CAA substrate. Furthermore, the patient’s normotensive status and absence of common PRES triggers, including eclampsia, cytotoxic drug exposure, or acute blood pressure elevation, strengthened the suspicion of CAA-RI. These features collectively favor CAA-RI over PRES, in which lesions are typically symmetric, posterior-predominant, and associated with identifiable precipitating factors.

In this context, the reasoning for this case is consistent with the Boston Criteria 2.0 [15], which supports a diagnosis of possible CAA in patients aged ≥50 years with appropriate clinical presentation and characteristic white matter changes on MRI, including a multispot pattern of bilaterally distributed subcortical fluid-attenuated inversion recovery (FLAIR) hyperintensities, as observed in our case, in the absence of an alternative cause.

Although the differential diagnosis included PRES, primary CNS vasculitis, autoimmune encephalitis, and infectious encephalitis. PRES was initially considered because of the vasogenic edema pattern without diffusion restriction. However, the asymmetric distribution of lesions, presence of cerebral microbleeds, normotensive status, and absence of typical triggers made this diagnosis less likely. Primary CNS vasculitis was considered, but the lack of systemic inflammatory activity, nonspecific CSF findings, and absence of imaging features suggestive of vessel wall inflammation argued against it. Autoimmune encephalitis was also less likely given the radiological pattern, absence of a typical limbic syndrome, and CSF profile without pleocytosis. Infectious causes, including neuroborreliosis and viral encephalitis, were excluded by microbiological testing, absence of intrathecal Borrelia antibody synthesis, and negative CSF PCR studies. Taken together, these findings supported CAA-RI as the most likely diagnosis.

This case highlights the importance of integrating clinical presentation and neuroimaging, and of excluding alternative etiologies. Early recognition allows timely immunosuppressive therapy, which may prevent irreversible neurological damage.

## Conclusions

CAA-RI should be considered as a possible diagnosis in elderly patients presenting with subacute cognitive decline and multifocal neurological deficits accompanied by white matter lesions and cerebral microbleeds. Because the clinical and radiological presentation may mimic infectious, neoplastic, or other inflammatory conditions, thorough diagnostic evaluation and careful interpretation of neuroimaging findings are essential. In this context, a structured diagnostic approach based on established criteria, such as the Boston criteria version 2.0, is essential for clinical decision-making. In particular, distinguishing CAA-RI from PRES is critical, as CAA-RI more often demonstrates asymmetric lesion distribution and is associated with cerebral microbleeds. In contrast, PRES typically presents with symmetric vasogenic edema and identifiable precipitating factors. 

Early recognition is particularly important because CAA-RI is a potentially treatable condition. Prompt initiation of corticosteroid therapy can lead to significant neurological recovery and radiological improvement. However, such treatment response should be interpreted in the context of the overall clinical and radiological findings. Greater awareness of this entity among clinicians may reduce diagnostic delays and improve clinical outcomes for affected patients.
